# Association of triglyceride and cholesterol with vestibular vertigo: Evidence from univariable and multivariable mendelian randomization and mediation analysis

**DOI:** 10.1016/j.bjorl.2025.101747

**Published:** 2026-01-07

**Authors:** Ying Mu, Yacheng Lu, Wei Fu

**Affiliations:** a Xi'an North Hospital, Department of Neurology, Shaanxi, China; b Air Fourth Medical University, Department of Anatomy, Histology and Embryology and K. K. Leung Brain Research Centre, Shaanxi, China; c Air Force Medical University, Xijing Hospital, Department of Geriatrics, Shaanxi, China

**Keywords:** Vertigo, Vestibular, Mendelian randomization, Triglyceride, Cholesterol

## Abstract

**Objectives:**

This study aimed to use Mendelian Randomization (MR) method to explore the potential causal association of triglyceride and cholesterol on the risk of vestibular vertigo and potential mediating factors.

**Methods:**

We extracted genetic variants associated with triglyceride and cholesterol from the genome-wide association study. Univariable two-sample MR were performed to evaluate the effects of triglyceride and cholesterol on the vestibular vertigo. Besides, Multivariate MR (MVMR) was used to estimate direct effects of triglyceride and cholesterol on the vestibular vertigo after adjusting for potential mediating factors. We also performed that a two-step mediation MR framework to elucidate potential mediators and to estimate the proportion of the association mediated.

**Results:**

Genetically predicted triglyceride and cholesterol were positively associated with the risk of vestibular vertigo. In the MVMR analysis, causal associations between triglyceride and cholesterol with vestibular vertigo remained stable and robust after adjusting for body mass index, hypertension, and type 2 diabetes, whereas the association between triglyceride and cholesterol and the risk of vestibular vertigo became nonsignificant after adjusting for vitamin D. In the mediation analysis, we found that the causal effect of cholesterol on vestibular vertigo might partly mediated by vitamin D.

**Conclusion:**

The present study provides robust evidence that genetically predicted triglyceride and cholesterol are independently causally associated with the risk of vestibular vertigo. Vitamin D also was found to mediate the causal pathways between cholesterol and vestibular vertigo.

**Level of evidence:**

Level III.

## Highlights

• Triglyceride and cholesterol are causally associated with vestibular vertigo.
• Vitamin D mediated the causal effect of cholesterol on vestibular vertigo.
• Vitamin D supplement may be helpful treatment of vestibular vertigo.

## Introduction

Vertigo and dizziness are common complaints of patients at all ages with a lifetime prevalence of around 15%–35% in the general population.^1^ Vertigo can be classified as peripheral or central vertigo, depending on dysfunctional locations in the vestibular pathway. Most of vertigo is peripheral vestibular (called vestibular vertigo), with the most common causes including Benign Paroxysmal Vertigo (BPV), Meniere’s Disease (MD), Vestibular Neuritis (VN), and Vestibular Dysfunction (VD).^1^ Individuals with vestibular vertigo report significantly diminished quality of life, with many requiring sick leave, giving up careers, and experiencing psychiatric and cognitive change.^2^ Vestibular vertigo are also amongst the most common complaints in older people, and are a growing public health concern since they put older people at a significantly higher risk of falling.^3^ Since the causes of vestibular vertigo are multi-factorial, it still remains unresolved for many patients. Thus, identifying risk factors and markers that are associated with vestibular vertigo would provide valuable insight into the disease and potential targets for intervention and prevention.

Observational studies have investigated several risk factors for vestibular vertigo, with dyslipidaemia being the most commonly recognized on.^4^^,^^5^ However, the findings of observational studies for dyslipidaemia have been inconsisten.^6^ Besides, observational studies are susceptible to confounding or reverse causation bias. Thus, the causal associations of dyslipidaemia with the risk of vestibular vertigo are still unclear. With this limited evidence from observational studies, Mendelian Randomization (MR) offers an opportunity to efficiently and reliably investigate the potential causal association between dyslipidaemia and vestibular vertigo. MR approach uses a genetic variant as the Instrumental Variables (IVs) in epidemiological studies to mimic a Randomized Controlled Trials (RCTs), where genetic alleles are randomly assorted at conception.^7^ This process is similar to the random allocation of treatment in RCTs and could therefore overcome the problems of reverse causation and confounding inherent in observational studies, a causal relationship is generally considered to be reliable. In this study, based on the MR approach, we conducted a two-sample MR study to examine the genetic correlation of triglyceride and cholesterol with vestibular vertigo. Additionally, Multivariate MR (MVMR) analyses was used to estimate independent effects of triglyceride and cholesterol on vestibular vertigo after adjusting for common risk factors, including Body Mass Index (BMI), hypertension, type 2 diabetes, and vitamin D. A mediation MR framework was additionally applied to elucidate potential mediators’ effects of these risk factors and to estimate the proportion of the association mediated.

## Methods

### Study design

**Fig. 1:**
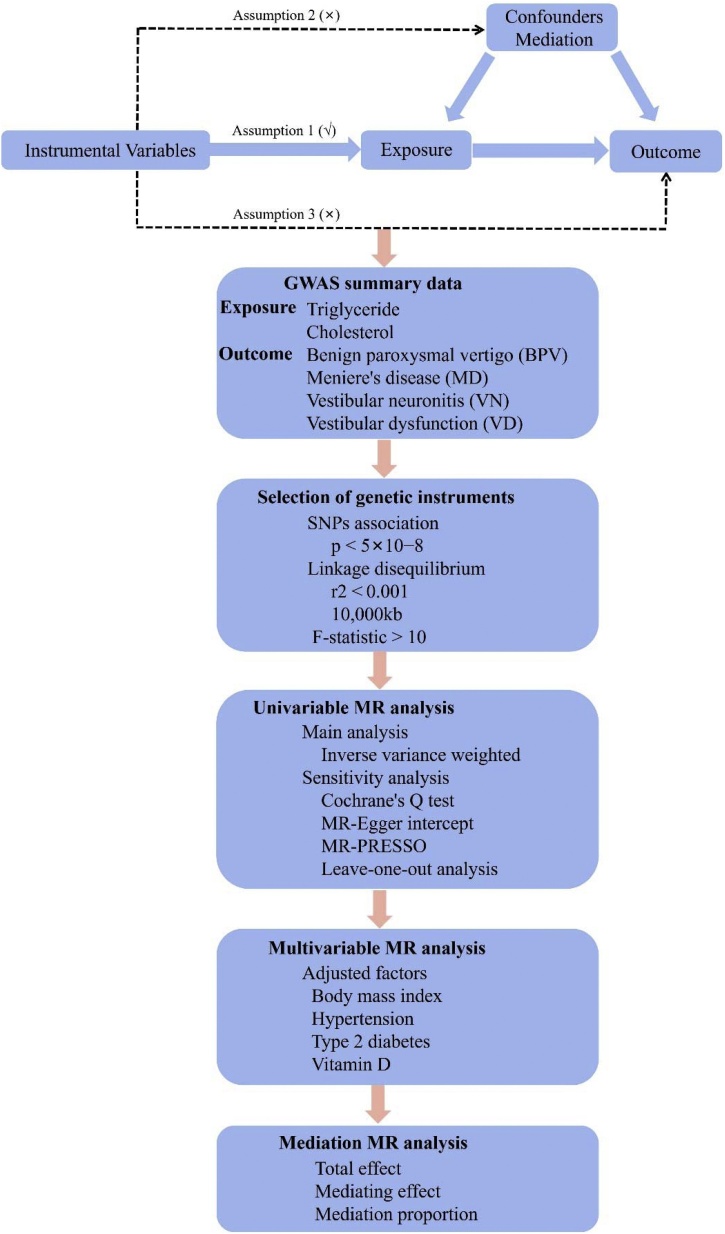
The graphical flow of the study design.

The study design of this study is depicted in Fig. 1, and we designed a MR study containing univariate and multivariate variables using published large, pooled data to examine the causal relationship of triglyceride and cholesterol on the risk of vestibular vertigo. The MR analysis included three core assumptions that need to be fulfilled: (1) The genetic variants are strongly associated with exposure (relevance); (2) The genetic variants are not related to the confounding factors of the exposure-outcome relationship (independence); and (3) The genetic variants do not affect the outcome through pathways other than the exposure (exclusion restriction). Then, a mediation MR framework was additionally applied to elucidate potential mediators that mediated the associations between triglyceride or cholesterol and vestibular vertigo and to estimate the proportion of the association mediated. The study’s use of publicly accessible data eliminated the need for ethical approval.

### Data source for and selection of genetic instruments

Our main exposure was genetically determined plasma lipids as instrumental variable from the Genome-Wide Association Study (GWAS) summary data, including triglyceride (n = 94,595) and cholesterol (n = 484,598).^8^^,^^9^ We selected Single Nucleotide Polymorphisms (SNPs) for exposures that exhibited a genome-wide significant association (p < 5 × 10^−8^) with the traits as IVs. Then, we estimated linkage disequilibrium among these SNPs based on the 1000 Genomes European reference panel.^10^ The candidate IVs for linkage disequilibrium (r^2^ > 0.001) and discarded variants that were within 1-Mb distance from other IVs with a stronger association. An F-statistic was calculated for each IVs to evaluate its association strength with exposure and included IVs with F-statistic >10.^11^ Finally, we harmonized the exposure and outcome datasets and eliminated palindromic SNPs.

Genetic associations with vestibular vertigo were obtained from the FinnGen study.^12^ The FinnGen study combines genetic data from nationwide biobanks and disease status from structured national healthcare databases. We obtained GWAS summary data of 4 vestibular vertigo from the latest FinnGen study R10 release, including BPV (9183 cases and 392,202 controls), MD (2977 cases and 392,202 controls), VN (2634 cases and 20,464 controls), and vestibular dysfunction (16,443 cases and 392,202 controls). Diagnosis of vestibular vertigo diseases are based on the international classification of diseases ICD-10.

The summary statistics for common risk factors were derived from the GWAS, including BMI (ukb-a-248, n = 336,107), hypertension (ukb-a-531, n = 337,199), type 2 diabetes (ebi-a-GCST90029024, n = 468,298), and vitamin D (ebi-a-GCST90014016, n = 373,045). All participants in this study are of European descent. Patients or the public WERE not involved in the design, or conduct, or reporting, or dissemination plans of our research. Detailed information about the data sources for each phenotype in our analysis is presented in Supplementary Table S1.

### Statistical analyses

For univariable two‐sample MR analysis, we used Inverse-Variance Weighted (IVW) methods as the primary analysis to assess the causal relationship of triglyceride and cholesterol on the risk of 4 vestibular vertigo. The inverse variance weighted estimate was used first, and the IVW used meta-analytic methods combined with the Wald estimates for each SNP to obtain a pooled causal estimate.^13^ Sensitivity analyses: Cochran’s *Q*-value and Q-derived p-values was employed to evaluate the heterogeneity among SNPs’ estimates.^14^ Once heterogeneity was found, the multiplicative random effects IVW method should be used for assessing the causal effect. We conducted an MR-Egger regression analysis to reveal possible horizontal pleiotropy by calculating the p-valve of its intercept.^15^ The MR-PRESSO (MR pleiotropy residual sum and outlier) test was employed, thus correcting for potential confounding factor.^16^ In order to eliminate the influence of a single SNP, we used the leave-one-out analysis to evaluate the robustness of the results. Additionally, a reverse two‐sample MR analysis was performed to explore if vestibular vertigo could causally increase the risk of triglyceride and cholesterol.

MVMR allows simultaneous assessment of the causal effects between multiple factors and outcome.^17^ Based on previous studies reported to be associated with vestibular vertigo, including BMI,^18^ hypertension,^4^^,^^5^ diabete,^4^^,^^19^ and vitamin D,^4^^,^^20^ these factors were considered as potential mediators or confounding in this study. We performed multivariable MR analyses with mutual adjustment to explore the independent effects of triglyceride and cholesterol on the risk of vestibular vertigo. For multivariable MR analysis, multivariable inverse variance weighted model was utilized as the primary analytical model.

We performed two-step MR to explore whether common risk factors have a mediation effect on the causal associations of triglyceride and cholesterol with the risk of vestibular vertigo. The specific method includes: (1) A causal correlation between exposure (triglyceride and cholesterol) and common risk factors. (2) Analyses of reverse causation of the common risk factors on triglyceride and cholesterol. If reverse causation is significant, this risk factors is confounding factors. If reverse causation is not significant, this risk factors is mediation factors. Then the causal effect of exposure on mediator (assume beta 1) are calculated. Use the same method to calculate the causal effect of mediator on vestibular vertigo (assume it is beta 2). beta1*beta2 can be used as the mediation effect from exposure to outcome.^21^^,^^22^ The total effect of an exposure on the outcome is estimated by univariable MR. The standard error of indirect effect was estimated using the delta method.^23^ The mediating proportion can also be calculated (mediation effect/total effect).

The Benjamini-Hochberg method that controls the False Discovery Rate (FDR) was applied to correct for multiple testing. The association with a Benjamini-Hochberg adjusted p-value <0.05 was deemed statistically significant. All analyses in this study were performed using R 4.3.0 software (R Foundation for Statistical Computing, Vienna, Austria). In different stages, packages including “TwoSample MR (version 0.5.6)”, “MR-PRESSO (version 1.0)”, and “Mendelian Randomization (version 0.9.0)” were used.

## Results

### Univariable two‐sample MR analysis for the causal association of triglyceride, cholesterol and vestibular vertigo

In the univariable MR analysis, after the above screening process, we selected 58, 60, 62, and 59 SNPs as the IVs for triglyceride to assess the associations between triglyceride and BPV, MD, VN, and VD, respectively. Besides, 67, 69, 61, and 67 SNPs were eventually obtained as the IVs for cholesterol to assess the associations between cholesterol and BPV, MD, VN, and VD, respectively. The F-statistics of all SNPs were more significant than 10, indicating that causal estimates were unlikely to be affected by weak instruments. Detailed information about the selected IVs are placed in the Supplementary Tables S2 and S3.

**Fig. 2:**
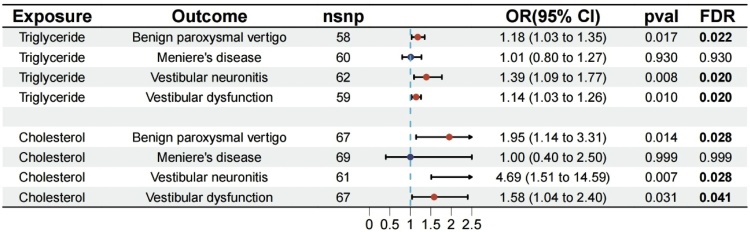
The causal effects of triglyceride and cholesterol on vestibular vertigo.

With the use of the inverse variance weighted method, we identified a causal relationship between triglyceride and vestibular vertigo. The causal effects of triglyceride on 4 vestibular vertigo using the IVW method are presented in Fig. 2. Genetically predicted triglyceride was positively associated with the risk of BPV, VN, and VD. These associations remained significant after FDR adjustment (OR = 1.18, 95% Confidence Interval [95% CI 1.03–1.35], p = 0.017, FDR = 0.022; OR = 1.39, 95% CI 1.09–1.77, p = 0.008, FDR = 0.020; OR = 1.14, 95% CI 1.03–1.26, p = 0.010, FDR = 0.020, respectively). However, we discovered no evidence that genetically predicted triglyceride was associated with the risk of MD (OR = 1.01, 95% CI 0.80–1.27, p = 0.93). Similarly, genetically predicted high cholesterol was associated with an increased risk of the vestibular vertigo after FDR adjustment of the p values including BPV (OR = 1.95, 95% CI 1.14–3.31; p = 0.014, FDR = 0.028), VN (OR = 4.69, 95% CI 1.51–14.59; p = 0.007, FDR = 0.028), VD (OR = 1.58, 95% CI 1.04–2.40; p = 0.031, FDR = 0.041). In contrast, no significant association was found between cholesterol and MD (OR = 1.0; 95% CI 0.40–2.50; p = 0.999). In the heterogeneity test, Cochran’s *Q* statistic was performed to test the heterogeneity. All results of Cochran’s *Q* test were above 0.05, signifying that there was no significant heterogeneity (Supplementary Table S4). The MR-PRESSO results showed no outlier SNPs. Moreover, the MR-Egger intercept test and the global test p-values both revealed no statistically significant results, suggesting no presence of horizontal pleiotropy (Supplementary Table S5). The leave-one-out plots further support the robustness of our results and suggest that the effects of any single SNP were unlikely to influence causal estimates (Supplementary Figs. S1 and S2).

### Multivariable MR analysis for the causal association of triglyceride, cholesterol and vestibular vertigo

**Fig. 3:**
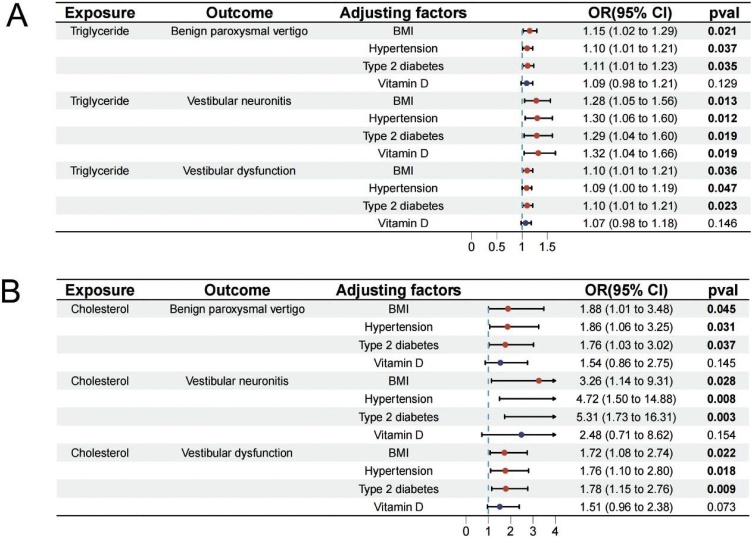
Multivariable mendelian randomization analyses. (A) The causal association of triglyceride and vestibular vertigo after adjusting risk factors; (B) The causal association of cholesterol and vestibular vertigo after adjusting risk factors.

In the MVMR framework, we adjusted for 4 potential factors (BMI, hypertension, type 2 diabetes, and vitamin D) to verify the independent associations of genetically predicted triglyceride and cholesterol with vestibular vertigo. We found that causal associations between triglyceride and cholesterol with vestibular vertigo remained stable and robust after adjusting for BMI, hypertension, and type 2 diabetes, whereas the association between triglyceride and cholesterol and the risk of BPV became nonsignificant after adjusting for vitamin D (OR = 1.09, 95% CI 0.98–1.21; p = 0.129; OR = 1.54, 95%CI 0.86–2.75; p = 0.145, Fig. 3, Supplementary Tables S6 and S7). Similarly, the association between triglyceride and cholesterol and the risk of VD became nonsignificant after adjusting for vitamin D (OR = 1.07, 95% CI 0.98–1.18; p = 0.146; OR = 1.51, 95% CI 0.96–2.38; p = 0.073), (Fig. 3, Supplementary Tables S6 and S7). Besides, the significant association between cholesterol and the risk of VN vanished after adjusting for vitamin D (OR = 2.48, 95% CI 0.71–8.62; p = 0.154, Fig. 3B, Supplementary Table S7).

### Mediation analysis for the causal association of triglyceride, cholesterol and vestibular vertigo

**Fig. 4:**
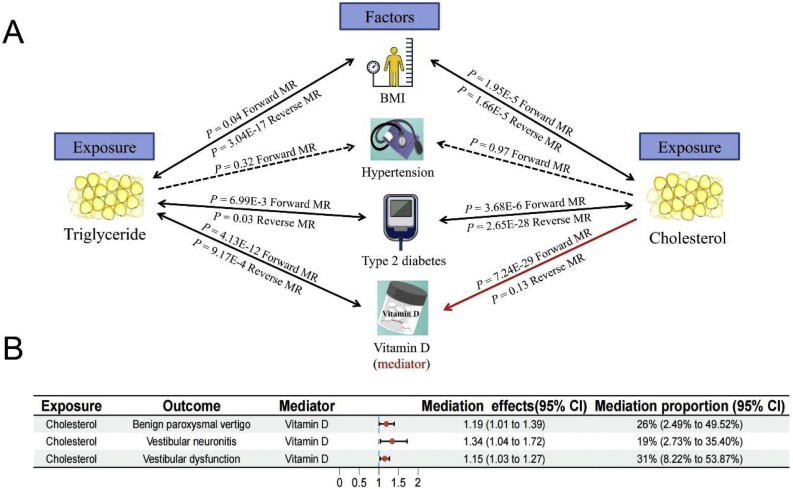
Mediation analysis for the causal association of triglyceride, cholesterol and vestibular vertigo. (A) Screening of mediators; (B) Forest map of mediation effect and mediating proportion.

In the current study, since triglyceride and cholesterol were no correlated with hypertension, hypertension were not performed to further mediation analyses (Fig. 4A, Supplementary Tables S8 and S9). Besides, we identified bidirectional causation of triglyceride and cholesterol on the BMI and type 2 diabetes. It was excluded from further mediation analyses (Fig. 4A, Supplementary Tables S8 and S9). Similarly, triglyceride was removed owning to evidence of bidirectional causation with vitamin D (Fig. 4A, Supplementary Table S8). Finally, we found that the causal effect of cholesterol on vestibular vertigo might partly mediated by vitamin D (Fig. 4A, Supplementary Table S9 and S10). Specifically, vitamin D explained 26% of the total effect of cholesterol on BPV. The proportion mediated by vitamin D in the associations between cholesterol and VN was 19%. And vitamin D mediate 31% effect of cholesterol on VD (Fig. 4B).

## Discussion

In the current study, leveraging large-scale genetic data, we mainly evaluated the associations between triglyceride and cholesterol and vestibular vertigo using univariable MR analysis, MVMR, and mediation analysis, which showed that genetically predicted triglyceride and cholesterol are independently causally associated with the risk of BPV, VN, and VD. Vitamin D was also found to be a mediator for the causal effects of cholesterol on vestibular vertigo. These findings offer new insights into the pathophysiological mechanisms by which dyslipidaemia contributes to vestibular vertigo and highlight the importance of vitamin D supplementation to prevent vestibular vertigo in patients with dyslipidaemia.

Our study indicates that genetically predicted triglyceride and cholesterol were associated with BPV, VN, and VD. Previous retrospective studies suggested that dyslipidaemia were associated with recurrence of BPV.^4^^,^^24^^,^^25^ Further analysis found that high cholesterol was important risk factors for the occurrence of BPV.^26^ Our MR study results supported these findings and further corroborated the causality of these associations. Besides, we also found that triglyceride has also been associated with increased risk of BPV. This finding has been reported rarely before. Consistence with our study, a large population-based study observed the comorbidities of patients with MD using a national population database and found that triglyceride and cholesterol might not be associated with the MD.^27^ In the previous studies, a correlation between VN and dyslipidemia was found, yet causality was not proven.^28^ Our MR results establish a causal relationship between VN and dyslipidemia. The correlation between levels of blood lipids and vestibular dysfunction has long been recognized.^29^ However, the study may be subject to reverse causation. This is because vestibular function stimulation has been demonstrated to confer beneficial effects on the blood lipid profiles.^30^ Besides, patients with vestibular dysfunction was found the significant increase in determining level of body fat.^31^ MR method avoided confounding or reverse causation bias. Thus, our study provides direct evidence that the triglyceride and cholesterol increased the risk of vestibular dysfunction. Our results and previous studies demonstrate that triglyceride and cholesterol might be an important role in the onset and progression of vestibular vertigo. A higher triglyceride and cholesterol level can cause vascular damage in the inner ear, which may lead to vestibular vertigo. Besides, higher triglyceride and cholesterol level may lead to early atherosclerotic plaque formation. This may trigger intravascular thrombosis and cause hypoperfusion of the vestibular organs.

Apart from triglyceride and cholesterol, risk factors for vestibular vertigo include BMI, hypertension, diabetes, vitamin D.^4^^,^^5^^,^^18^, ^19^, ^20^^,^^28^ Furthermore, triglyceride and cholesterol were also associated with BMI, hypertension, diabetes, and vitamin D.^32^, ^33^, ^34^, ^35^ These factors may affect the occurrence of vestibular vertigo. In order to investigate independent causal associations between triglyceride and cholesterol with vestibular vertigo. We then conducted multivariable MR analyses. MVMR allows simultaneous assessment of relevant exposures by incorporating genetic variants of multiple risk factors into the same model to minimize the impact of confounding variables. It is worth highlighting that using MVMR methods in this study was to assess whether triglyceride and cholesterol have an direct impact on vestibular vertigo independently of BMI, hypertension, type 2 diabetes, and vitamin D. After adjusting for BMI, hypertension, and type 2 diabetes, we found that triglyceride and cholesterol are still causally associated with the risk of BPV, VN, and VD. But this relationship became nonsignificant when vitamin D was considered in a multivariable model. These results showed that vitamin D seemed to partly mediate the associations of triglyceride and cholesterol with vestibular vertigo, which indicates that vitamin D may be involved in the pathological process of vitamin D. Indeed, vitamin D deficiency and dyslipidaemia are a common comorbidity observed in individuals with vestibular vertigo.^36^^,^^37^ The vitamin D is essential for maintaining the homeostasis of vestibular system. Low vitamin D levels result in disruption of the calcium absorptive system in the inner ear, increasing receptor activator of nuclear factor-kappa B ligand with decreased osteoprotegrin in the bone, causing resorption of calcium carbonate in the otoconia and increased osteoclast differentiation and calcium phosphate resorption, respectively.^38^ Resorption of calcium (demineralization) results in fragmentation of otoconia and increased vulnerability to vestibular vertigo.^39^ Besides, vitamin D could affect vestibular function via vitamin D related signal pathways and vitamin D receptors system is the most important in this signaling pathway. Study have detected robust vitamin D receptors expression in the vestibular organ and a mutation mice in vitamin D receptors showed several vestibular dysfunctions including shorter latency to fall from the rotarod, smaller fall angle in the tilting box test, and aberrant poor swimming. This basic science evidence suggests that normal vitamin D signaling is required in balance control and thus normal serum concentrations are required to maintain postural stability and balance.^40^ Moreover, vitamin D receptors deficiency found to induce vertigo.^41^ Therefore, any factor that affects vitamin D would be associated with the risk of vestibular vertigo. Previous studies have shown a relationship between vitamin D and blood lipids. Lupton et al. showed that vitamin D was negatively correlated with cholesterol in adults.^42^ Another study found that elevated serum triglyceride evels was causally associated with vitamin D deficiency.^43^

Although there was still a lack of comprehensive interpretation by which vitamin D affects lipid levels, there are still some studies exploring the potential mechanism. On the one hand, diet-induced elevation of circulating cholesterol could reduce vitamin D levels by suppressing hepatic CYP2R1 expression.^44^ On the other hand, there is a decrease in gene expression of vitamin D metabolizing enzymes in human adipose tissue.^45^ It may be involved in the development of vitamin D deficiency. After a stringent screening of causal mediators, we found that vitamin D seemed to partly mediate the associations of cholesterol with vestibular vertigo. We further calculated the mediating proportion of vitamin D between cholesterol and vestibular vertigo. Specifically, vitamin D mediate 26%, 19%, and 31% effect of cholesterol on BPV, VN, and VD, respectively. These findings highlight the potential of targeted of triglyceride, cholesterol, and vitamin D as a therapeutic strategy for dyslipidaemia patients with vestibular vertigo. Interestingly, a RCT study found that vitamin D supplementation in BPV patients with deficiency or insufficiency decreases both the numbers of relapsing patients and relapses per patient.^20^ Subgroup analyses showed that the preventive effect of vitamin D was related to obesity.^20^ Hyperlipidemia is commonly seen with obesity. We speculate that patients with hyperlipidemia may be involved in the development of vitamin D deficiency. Further study is needed to determine whether treating obesity or hyperlipidemia can prevent BPV.

Several limitations should also be noted that our study is limited to individuals of European ancestry, and further investigation is necessary to validate our findings across other ancestries. Second, this study was performed based on summary-level statistics. We can neither explore the nonlinear relationship between triglyceride and cholesterol and vestibular vertigo. Third, summary-level GWAS data precluded subgroup analyses of age and gender. We were unable to explore age and gender specific associations.

## Conclusions

In conclusion, this study provides robust evidence that genetically predicted triglyceride and cholesterol are independently causally associated with the risk of BPV, VN, and VD. Furthermore, vitamin D were found to mediate the causal pathways between cholesterol and vestibular vertigo, and vitamin D supplement may be helpful in terms of vestibular vertigo prevention in patients with dyslipidaemia.

## ORCID ID

Ying Mu: 0000-0001-6409-2846

Yacheng Lu: 0000-0002-6198-9567

Wei Fu: 0000-0003-3594-8785

## Funding

This study was supported by the 10.13039/501100001809National Natural Science Foundation of China (No. 82202788), The Key R&D Program of Shaanxi Province (No. 2022SF-283), and the National Natural Science Foundation of Shaanxi (No. 2023-JC-YB-751).

## Data availability statement

We declare that all data are available in repository.

## Declaration of competing interest

The authors declare that the research was conducted in the absence of any commercial or financial relationships that could be construed as a potential conflict of interest.
