# Supplementary material for: Association of triglyceride and cholesterol with vestibular vertigo: Evidence from univariable and multivariable mendelian randomization and mediation analysis
Source: Braz J Otorhinolaryngol. 2026 Jan 7;92(2):101747. doi: 10.1016/j.bjorl.2025.101747 (PMC12813604; doi:10.1016/j.bjorl.2025.101747)

**BJORL-D-24-00372_Supplementary Material**

**Supplementary Table 1** GWAS data sources in this MR study.

| **Phenotype** | **Data source** | **Phenotypic code** | **Sample size** | **Ancestry** |
| --- | --- | --- | --- | --- |
| Triglyceride | Willer CJ.et al. | ebi-a-GCST002216 | 94.595 | European |
| Cholesterol | Dönertaş HM. et al. | ebi-a-GCST90038690 | 484.598 | European |
| Benign paroxysmal vertigo | FinnGen | H8_BPV | 401.385 | European |
| Meniere's disease | FinnGen | H8_MENIERE | 395.179 | European |
| Vestibular neuronitis | FinnGen | H8_VESTIBNEUR | 394.836 | European |
| Vestibular dysfunction | FinnGen | H8_VERTIGO | 408.645 | European |
| Body mass index | Neale Lab | ukb-a-248 | 336.107 | European |
| Hypertension | Neale Lab | ukb-a-531 | 337.199 | European |
| Type 2 diabetes | Loh PR.et al. | ebi-a-GCST90029024 | 484.598 | European |
| Vitamin D | Mbatchou J.et al. | ebi-a-GCST90014016 | 373.045 | European |

**Supplementary Table 2** Genetic instruments screened by GWAS to be associated with triglyceride on vestibular vertigo.

| **Exposure** | **Outcome** | **SNP** | **EA** | **OA** | **F-statistics** | **Beta** | **SE** | **p-value** |
| --- | --- | --- | --- | --- | --- | --- | --- | --- |
| **Triglyceride** | **BPV** | rs10426094 | T | C | 36.30 | -0.0241 | 0.004 | 1.69E-09 |
|  |  | rs10440120 | A | C | 48.37 | -0.0306 | 0.0044 | 3.54E-12 |
|  |  | rs10501321 | C | T | 38.09 | -0.0216 | 0.0035 | 6.77E-10 |
|  |  | rs10513688 | A | G | 29.86 | 0.0306 | 0.0056 | 4.65E-08 |
|  |  | rs1077834 | C | T | 131.41 | 0.047 | 0.0041 | 1.00E-200 |
|  |  | rs10861661 | C | A | 30.65 | 0.0227 | 0.0041 | 3.08E-08 |
|  |  | rs11057408 | T | G | 54.34 | -0.0258 | 0.0035 | 1.69E-13 |
|  |  | rs11204072 | C | T | 74.19 | -0.0379 | 0.0044 | 1.00E-200 |
|  |  | rs11613352 | T | C | 51.55 | -0.028 | 0.0039 | 7.00E-13 |
|  |  | rs11784251 | G | A | 68.98 | -0.0299 | 0.0036 | 1.00E-200 |
|  |  | rs117935983 | G | T | 101.28 | 0.1731 | 0.0172 | 1.00E-200 |
|  |  | rs1211644 | C | T | 31.61 | -0.0298 | 0.0053 | 1.88E-08 |
|  |  | rs12446515 | T | C | 69.22 | -0.0416 | 0.005 | 1.00E-200 |
|  |  | rs12602912 | T | C | 34.55 | 0.0241 | 0.0041 | 4.15E-09 |
|  |  | rs12748152 | T | C | 39.75 | 0.0372 | 0.0059 | 2.88E-10 |
|  |  | rs13389219 | T | C | 63.53 | -0.0271 | 0.0034 | 1.55E-15 |
|  |  | rs1688030 | C | T | 30.90 | 0.0378 | 0.0068 | 2.72E-08 |
|  |  | rs17005886 | G | A | 112.36 | -0.0795 | 0.0075 | 1.00E-200 |
|  |  | rs17134533 | A | G | 51.84 | -0.0324 | 0.0045 | 6.02E-13 |
|  |  | rs174528 | C | T | 164.44 | 0.0436 | 0.0034 | 1.00E-200 |
|  |  | rs17513135 | T | C | 31.82 | 0.022 | 0.0039 | 1.69E-08 |
|  |  | rs1883025 | T | C | 29.98 | -0.0219 | 0.004 | 4.38E-08 |
|  |  | rs2068888 | A | G | 50.24 | -0.0241 | 0.0034 | 1.36E-12 |
|  |  | rs2250802 | A | G | 38.64 | 0.023 | 0.0037 | 5.09E-10 |
|  |  | rs236996 | A | G | 70.56 | -0.0294 | 0.0035 | 1.00E-200 |
|  |  | rs2384629 | G | A | 95.03 | -0.1277 | 0.0131 | 1.00E-200 |
|  |  | rs2385114 | T | C | 130.34 | 0.0411 | 0.0036 | 1.00E-200 |
|  |  | rs2665357 | C | A | 41.27 | 0.0212 | 0.0033 | 1.33E-10 |
|  |  | rs287621 | C | T | 36.00 | -0.0222 | 0.0037 | 1.97E-09 |
|  |  | rs2925979 | C | T | 32.43 | -0.0205 | 0.0036 | 1.24E-08 |
|  |  | rs2943645 | T | C | 67.34 | 0.0279 | 0.0034 | 2.22E-16 |
|  |  | rs3198697 | T | C | 33.91 | -0.0198 | 0.0034 | 5.76E-09 |
|  |  | rs35242582 | G | A | 61.41 | 0.0431 | 0.0055 | 4.66E-15 |
|  |  | rs3761445 | A | G | 46.56 | 0.0232 | 0.0034 | 8.88E-12 |
|  |  | rs3793243 | G | A | 31.53 | -0.0219 | 0.0039 | 1.96E-08 |
|  |  | rs3843467 | T | G | 71.44 | 0.0355 | 0.0042 | 1.00E-200 |
|  |  | rs38855 | G | A | 32.11 | -0.0187 | 0.0033 | 1.46E-08 |
|  |  | rs454748 | A | G | 33.31 | 0.0202 | 0.0035 | 7.86E-09 |
|  |  | rs4666040 | G | A | 45.65 | -0.025 | 0.0037 | 1.41E-11 |
|  |  | rs4704727 | G | T | 68.18 | 0.0289 | 0.0035 | 2.22E-16 |
|  |  | rs4719841 | G | A | 46.56 | 0.0232 | 0.0034 | 8.88E-12 |
|  |  | rs4810479 | T | C | 155.59 | -0.0474 | 0.0038 | 1.00E-200 |
|  |  | rs4846913 | A | C | 93.16 | -0.0444 | 0.0046 | 1.00E-200 |
|  |  | rs516226 | T | C | 106.25 | 0.067 | 0.0065 | 1.00E-200 |
|  |  | rs634869 | C | T | 67.94 | -0.0272 | 0.0033 | 2.22E-16 |
|  |  | rs645040 | T | G | 53.66 | 0.0293 | 0.004 | 2.39E-13 |
|  |  | rs6908994 | T | C | 69.69 | 0.0384 | 0.0046 | 1.00E-200 |
|  |  | rs6968554 | G | A | 33.31 | 0.0202 | 0.0035 | 7.86E-09 |
|  |  | rs6995541 | G | A | 51.30 | 0.0265 | 0.0037 | 7.94E-13 |
|  |  | rs7033354 | T | C | 31.23 | -0.019 | 0.0034 | 2.29E-08 |
|  |  | rs719726 | T | C | 32.33 | 0.0199 | 0.0035 | 1.30E-08 |
|  |  | rs731839 | A | G | 38.72 | -0.0224 | 0.0036 | 4.90E-10 |
|  |  | rs749671 | A | G | 38.51 | -0.0211 | 0.0034 | 5.44E-10 |
|  |  | rs77917292 | A | G | 72.03 | 0.1341 | 0.0158 | 1.00E-200 |
|  |  | rs7897379 | C | T | 68.94 | -0.0274 | 0.0033 | 1.00E-200 |
|  |  | rs78980904 | C | T | 118.97 | -0.0589 | 0.0054 | 1.00E-200 |
|  |  | rs8077889 | C | A | 36.00 | 0.0252 | 0.0042 | 1.97E-09 |
|  |  | rs998584 | A | C | 62.71 | 0.0293 | 0.0037 | 2.44E-15 |
| **Triglyceride** | **MD** |  |  |  |  |  |  |  |
|  |  | rs10426094 | T | C | 36.30 | -0.0241 | 0.004 | 1.69E-09 |
|  |  | rs10440120 | A | C | 48.37 | -0.0306 | 0.0044 | 3.54E-12 |
|  |  | rs10501321 | C | T | 38.09 | -0.0216 | 0.0035 | 6.77E-10 |
|  |  | rs10513688 | A | G | 29.86 | 0.0306 | 0.0056 | 4.65E-08 |
|  |  | rs1077834 | C | T | 131.41 | 0.047 | 0.0041 | 1.00E-200 |
|  |  | rs10861661 | C | A | 30.65 | 0.0227 | 0.0041 | 3.08E-08 |
|  |  | rs11057408 | T | G | 54.34 | -0.0258 | 0.0035 | 1.69E-13 |
|  |  | rs11204072 | C | T | 74.19 | -0.0379 | 0.0044 | 1.00E-200 |
|  |  | rs11613352 | T | C | 51.55 | -0.028 | 0.0039 | 7.00E-13 |
|  |  | rs11784251 | G | A | 68.98 | -0.0299 | 0.0036 | 1.00E-200 |
|  |  | rs117935983 | G | T | 101.28 | 0.1731 | 0.0172 | 1.00E-200 |
|  |  | rs1211644 | C | T | 31.61 | -0.0298 | 0.0053 | 1.88E-08 |
|  |  | rs12446515 | T | C | 69.22 | -0.0416 | 0.005 | 1.00E-200 |
|  |  | rs12602912 | T | C | 34.55 | 0.0241 | 0.0041 | 4.15E-09 |
|  |  | rs13389219 | T | C | 63.53 | -0.0271 | 0.0034 | 1.55E-15 |
|  |  | rs1341267 | A | C | 31.09 | -0.0184 | 0.0033 | 2.46E-08 |
|  |  | rs1688030 | C | T | 30.90 | 0.0378 | 0.0068 | 2.72E-08 |
|  |  | rs17005886 | G | A | 112.36 | -0.0795 | 0.0075 | 1.00E-200 |
|  |  | rs17134533 | A | G | 51.84 | -0.0324 | 0.0045 | 6.02E-13 |
|  |  | rs174528 | C | T | 164.44 | 0.0436 | 0.0034 | 1.00E-200 |
|  |  | rs17513135 | T | C | 31.82 | 0.022 | 0.0039 | 1.69E-08 |
|  |  | rs1883025 | T | C | 29.98 | -0.0219 | 0.004 | 4.38E-08 |
|  |  | rs2068888 | A | G | 50.24 | -0.0241 | 0.0034 | 1.36E-12 |
|  |  | rs2250802 | A | G | 38.64 | 0.023 | 0.0037 | 5.09E-10 |
|  |  | rs236996 | A | G | 70.56 | -0.0294 | 0.0035 | 1.00E-200 |
|  |  | rs2384629 | G | A | 95.03 | -0.1277 | 0.0131 | 1.00E-200 |
|  |  | rs2385114 | T | C | 130.34 | 0.0411 | 0.0036 | 1.00E-200 |
|  |  | rs2665357 | C | A | 41.27 | 0.0212 | 0.0033 | 1.33E-10 |
|  |  | rs287621 | C | T | 36.00 | -0.0222 | 0.0037 | 1.97E-09 |
|  |  | rs2925979 | C | T | 32.43 | -0.0205 | 0.0036 | 1.24E-08 |
|  |  | rs2943645 | T | C | 67.34 | 0.0279 | 0.0034 | 2.22E-16 |
|  |  | rs3198697 | T | C | 33.91 | -0.0198 | 0.0034 | 5.76E-09 |
|  |  | rs35242582 | G | A | 61.41 | 0.0431 | 0.0055 | 4.66E-15 |
|  |  | rs3761445 | A | G | 46.56 | 0.0232 | 0.0034 | 8.88E-12 |
|  |  | rs3793243 | G | A | 31.53 | -0.0219 | 0.0039 | 1.96E-08 |
|  |  | rs3843467 | T | G | 71.44 | 0.0355 | 0.0042 | 1.00E-200 |
|  |  | rs38855 | G | A | 32.11 | -0.0187 | 0.0033 | 1.46E-08 |
|  |  | rs454748 | A | G | 33.31 | 0.0202 | 0.0035 | 7.86E-09 |
|  |  | rs4666040 | G | A | 45.65 | -0.025 | 0.0037 | 1.41E-11 |
|  |  | rs4704727 | G | T | 68.18 | 0.0289 | 0.0035 | 2.22E-16 |
|  |  | rs4719841 | G | A | 46.56 | 0.0232 | 0.0034 | 8.88E-12 |
|  |  | rs4804311 | G | A | 42.68 | -0.0392 | 0.006 | 6.43E-11 |
|  |  | rs4810479 | T | C | 155.59 | -0.0474 | 0.0038 | 1.00E-200 |
|  |  | rs4846913 | A | C | 93.16 | -0.0444 | 0.0046 | 1.00E-200 |
|  |  | rs492571 | C | T | 82.44 | 0.0799 | 0.0088 | 1.00E-200 |
|  |  | rs516226 | T | C | 106.25 | 0.067 | 0.0065 | 1.00E-200 |
|  |  | rs634869 | C | T | 67.94 | -0.0272 | 0.0033 | 2.22E-16 |
|  |  | rs645040 | T | G | 53.66 | 0.0293 | 0.004 | 2.39E-13 |
|  |  | rs6831256 | G | A | 54.34 | 0.0258 | 0.0035 | 1.69E-13 |
|  |  | rs6908994 | T | C | 69.69 | 0.0384 | 0.0046 | 1.00E-200 |
|  |  | rs6968554 | G | A | 33.31 | 0.0202 | 0.0035 | 7.86E-09 |
|  |  | rs6995541 | G | A | 51.30 | 0.0265 | 0.0037 | 7.94E-13 |
|  |  | rs7033354 | T | C | 31.23 | -0.019 | 0.0034 | 2.29E-08 |
|  |  | rs719726 | T | C | 32.33 | 0.0199 | 0.0035 | 1.30E-08 |
|  |  | rs731839 | A | G | 38.72 | -0.0224 | 0.0036 | 4.90E-10 |
|  |  | rs749671 | A | G | 38.51 | -0.0211 | 0.0034 | 5.44E-10 |
|  |  | rs77917292 | A | G | 72.03 | 0.1341 | 0.0158 | 1.00E-200 |
|  |  | rs78980904 | C | T | 118.97 | -0.0589 | 0.0054 | 1.00E-200 |
|  |  | rs8077889 | C | A | 36.00 | 0.0252 | 0.0042 | 1.97E-09 |
|  |  | rs998584 | A | C | 62.71 | 0.0293 | 0.0037 | 2.44E-15 |
| **Triglyceride** | **VN** |  |  |  |  |  |  |  |
|  |  | rs10426094 | T | C | 36.30 | -0.0241 | 0.004 | 1.69E-09 |
|  |  | rs10440120 | A | C | 48.37 | -0.0306 | 0.0044 | 3.54E-12 |
|  |  | rs10501321 | C | T | 38.09 | -0.0216 | 0.0035 | 6.77E-10 |
|  |  | rs10513688 | A | G | 29.86 | 0.0306 | 0.0056 | 4.65E-08 |
|  |  | rs1077834 | C | T | 131.41 | 0.047 | 0.0041 | 1.00E-200 |
|  |  | rs10861661 | C | A | 30.65 | 0.0227 | 0.0041 | 3.08E-08 |
|  |  | rs11057408 | T | G | 54.34 | -0.0258 | 0.0035 | 1.69E-13 |
|  |  | rs11204072 | C | T | 74.19 | -0.0379 | 0.0044 | 1.00E-200 |
|  |  | rs11613352 | T | C | 51.55 | -0.028 | 0.0039 | 7.00E-13 |
|  |  | rs11784251 | G | A | 68.98 | -0.0299 | 0.0036 | 1.00E-200 |
|  |  | rs117935983 | G | T | 101.28 | 0.1731 | 0.0172 | 1.00E-200 |
|  |  | rs1211644 | C | T | 31.61 | -0.0298 | 0.0053 | 1.88E-08 |
|  |  | rs12446515 | T | C | 69.22 | -0.0416 | 0.005 | 1.00E-200 |
|  |  | rs12602912 | T | C | 34.55 | 0.0241 | 0.0041 | 4.15E-09 |
|  |  | rs12748152 | T | C | 39.75 | 0.0372 | 0.0059 | 2.88E-10 |
|  |  | rs13389219 | T | C | 63.53 | -0.0271 | 0.0034 | 1.55E-15 |
|  |  | rs1341267 | A | C | 31.09 | -0.0184 | 0.0033 | 2.46E-08 |
|  |  | rs1688030 | C | T | 30.90 | 0.0378 | 0.0068 | 2.72E-08 |
|  |  | rs17005886 | G | A | 112.36 | -0.0795 | 0.0075 | 1.00E-200 |
|  |  | rs17134533 | A | G | 51.84 | -0.0324 | 0.0045 | 6.02E-13 |
|  |  | rs174528 | C | T | 164.44 | 0.0436 | 0.0034 | 1.00E-200 |
|  |  | rs17513135 | T | C | 31.82 | 0.022 | 0.0039 | 1.69E-08 |
|  |  | rs1883025 | T | C | 29.98 | -0.0219 | 0.004 | 4.38E-08 |
|  |  | rs2068888 | A | G | 50.24 | -0.0241 | 0.0034 | 1.36E-12 |
|  |  | rs2250802 | A | G | 38.64 | 0.023 | 0.0037 | 5.09E-10 |
|  |  | rs236996 | A | G | 70.56 | -0.0294 | 0.0035 | 1.00E-200 |
|  |  | rs2384629 | G | A | 95.03 | -0.1277 | 0.0131 | 1.00E-200 |
|  |  | rs2385114 | T | C | 130.34 | 0.0411 | 0.0036 | 1.00E-200 |
|  |  | rs2665357 | C | A | 41.27 | 0.0212 | 0.0033 | 1.33E-10 |
|  |  | rs287621 | C | T | 36.00 | -0.0222 | 0.0037 | 1.97E-09 |
|  |  | rs2925979 | C | T | 32.43 | -0.0205 | 0.0036 | 1.24E-08 |
|  |  | rs2943645 | T | C | 67.34 | 0.0279 | 0.0034 | 2.22E-16 |
|  |  | rs3198697 | T | C | 33.91 | -0.0198 | 0.0034 | 5.76E-09 |
|  |  | rs35242582 | G | A | 61.41 | 0.0431 | 0.0055 | 4.66E-15 |
|  |  | rs3761445 | A | G | 46.56 | 0.0232 | 0.0034 | 8.88E-12 |
|  |  | rs3793243 | G | A | 31.53 | -0.0219 | 0.0039 | 1.96E-08 |
|  |  | rs3843467 | T | G | 71.44 | 0.0355 | 0.0042 | 1.00E-200 |
|  |  | rs38855 | G | A | 32.11 | -0.0187 | 0.0033 | 1.46E-08 |
|  |  | rs454748 | A | G | 33.31 | 0.0202 | 0.0035 | 7.86E-09 |
|  |  | rs4666040 | G | A | 45.65 | -0.025 | 0.0037 | 1.41E-11 |
|  |  | rs4704727 | G | T | 68.18 | 0.0289 | 0.0035 | 2.22E-16 |
|  |  | rs4719841 | G | A | 46.56 | 0.0232 | 0.0034 | 8.88E-12 |
|  |  | rs4804311 | G | A | 42.68 | -0.0392 | 0.006 | 6.43E-11 |
|  |  | rs4810479 | T | C | 155.59 | -0.0474 | 0.0038 | 1.00E-200 |
|  |  | rs4846913 | A | C | 93.16 | -0.0444 | 0.0046 | 1.00E-200 |
|  |  | rs492571 | C | T | 82.44 | 0.0799 | 0.0088 | 1.00E-200 |
|  |  | rs516226 | T | C | 106.25 | 0.067 | 0.0065 | 1.00E-200 |
|  |  | rs634869 | C | T | 67.94 | -0.0272 | 0.0033 | 2.22E-16 |
|  |  | rs645040 | T | G | 53.66 | 0.0293 | 0.004 | 2.39E-13 |
|  |  | rs6831256 | G | A | 54.34 | 0.0258 | 0.0035 | 1.69E-13 |
|  |  | rs6908994 | T | C | 69.69 | 0.0384 | 0.0046 | 1.00E-200 |
|  |  | rs6968554 | G | A | 33.31 | 0.0202 | 0.0035 | 7.86E-09 |
|  |  | rs6995541 | G | A | 51.30 | 0.0265 | 0.0037 | 7.94E-13 |
|  |  | rs7033354 | T | C | 31.23 | -0.019 | 0.0034 | 2.29E-08 |
|  |  | rs719726 | T | C | 32.33 | 0.0199 | 0.0035 | 1.30E-08 |
|  |  | rs731839 | A | G | 38.72 | -0.0224 | 0.0036 | 4.90E-10 |
|  |  | rs749671 | A | G | 38.51 | -0.0211 | 0.0034 | 5.44E-10 |
|  |  | rs77917292 | A | G | 72.03 | 0.1341 | 0.0158 | 1.00E-200 |
|  |  | rs7897379 | C | T | 68.94 | -0.0274 | 0.0033 | 1.00E-200 |
|  |  | rs78980904 | C | T | 118.97 | -0.0589 | 0.0054 | 1.00E-200 |
|  |  | rs8077889 | C | A | 36.00 | 0.0252 | 0.0042 | 1.97E-09 |
|  |  | rs998584 | A | C | 62.71 | 0.0293 | 0.0037 | 2.44E-15 |
| **Triglyceride** | **VD** |  |  |  |  |  |  |  |
|  |  | rs10426094 | T | C | 36.30 | -0.0241 | 0.004 | 1.69E-09 |
|  |  | rs10440120 | A | C | 48.37 | -0.0306 | 0.0044 | 3.54E-12 |
|  |  | rs10501321 | C | T | 38.09 | -0.0216 | 0.0035 | 6.77E-10 |
|  |  | rs10513688 | A | G | 29.86 | 0.0306 | 0.0056 | 4.65E-08 |
|  |  | rs1077834 | C | T | 131.41 | 0.047 | 0.0041 | 1.00E-200 |
|  |  | rs11057408 | T | G | 54.34 | -0.0258 | 0.0035 | 1.69E-13 |
|  |  | rs11204072 | C | T | 74.19 | -0.0379 | 0.0044 | 1.00E-200 |
|  |  | rs11613352 | T | C | 51.55 | -0.028 | 0.0039 | 7.00E-13 |
|  |  | rs11784251 | G | A | 68.98 | -0.0299 | 0.0036 | 1.00E-200 |
|  |  | rs117935983 | G | T | 101.28 | 0.1731 | 0.0172 | 1.00E-200 |
|  |  | rs1211644 | C | T | 31.61 | -0.0298 | 0.0053 | 1.88E-08 |
|  |  | rs12446515 | T | C | 69.22 | -0.0416 | 0.005 | 1.00E-200 |
|  |  | rs12602912 | T | C | 34.55 | 0.0241 | 0.0041 | 4.15E-09 |
|  |  | rs12748152 | T | C | 39.75 | 0.0372 | 0.0059 | 2.88E-10 |
|  |  | rs13389219 | T | C | 63.53 | -0.0271 | 0.0034 | 1.55E-15 |
|  |  | rs1688030 | C | T | 30.90 | 0.0378 | 0.0068 | 2.72E-08 |
|  |  | rs17005886 | G | A | 112.36 | -0.0795 | 0.0075 | 1.00E-200 |
|  |  | rs17134533 | A | G | 51.84 | -0.0324 | 0.0045 | 6.02E-13 |
|  |  | rs174528 | C | T | 164.44 | 0.0436 | 0.0034 | 1.00E-200 |
|  |  | rs17513135 | T | C | 31.82 | 0.022 | 0.0039 | 1.69E-08 |
|  |  | rs1883025 | T | C | 29.98 | -0.0219 | 0.004 | 4.38E-08 |
|  |  | rs2068888 | A | G | 50.24 | -0.0241 | 0.0034 | 1.36E-12 |
|  |  | rs2250802 | A | G | 38.64 | 0.023 | 0.0037 | 5.09E-10 |
|  |  | rs236996 | A | G | 70.56 | -0.0294 | 0.0035 | 1.00E-200 |
|  |  | rs2384629 | G | A | 95.03 | -0.1277 | 0.0131 | 1.00E-200 |
|  |  | rs2385114 | T | C | 130.34 | 0.0411 | 0.0036 | 1.00E-200 |
|  |  | rs2665357 | C | A | 41.27 | 0.0212 | 0.0033 | 1.33E-10 |
|  |  | rs287621 | C | T | 36.00 | -0.0222 | 0.0037 | 1.97E-09 |
|  |  | rs2925979 | C | T | 32.43 | -0.0205 | 0.0036 | 1.24E-08 |
|  |  | rs2943645 | T | C | 67.34 | 0.0279 | 0.0034 | 2.22E-16 |
|  |  | rs3198697 | T | C | 33.91 | -0.0198 | 0.0034 | 5.76E-09 |
|  |  | rs35242582 | G | A | 61.41 | 0.0431 | 0.0055 | 4.66E-15 |
|  |  | rs3761445 | A | G | 46.56 | 0.0232 | 0.0034 | 8.88E-12 |
|  |  | rs3793243 | G | A | 31.53 | -0.0219 | 0.0039 | 1.96E-08 |
|  |  | rs3843467 | T | G | 71.44 | 0.0355 | 0.0042 | 1.00E-200 |
|  |  | rs38855 | G | A | 32.11 | -0.0187 | 0.0033 | 1.46E-08 |
|  |  | rs454748 | A | G | 33.31 | 0.0202 | 0.0035 | 7.86E-09 |
|  |  | rs4666040 | G | A | 45.65 | -0.025 | 0.0037 | 1.41E-11 |
|  |  | rs4704727 | G | T | 68.18 | 0.0289 | 0.0035 | 2.22E-16 |
|  |  | rs4719841 | G | A | 46.56 | 0.0232 | 0.0034 | 8.88E-12 |
|  |  | rs4804311 | G | A | 42.68 | -0.0392 | 0.006 | 6.43E-11 |
|  |  | rs4810479 | T | C | 155.59 | -0.0474 | 0.0038 | 1.00E-200 |
|  |  | rs4846913 | A | C | 93.16 | -0.0444 | 0.0046 | 1.00E-200 |
|  |  | rs516226 | T | C | 106.25 | 0.067 | 0.0065 | 1.00E-200 |
|  |  | rs634869 | C | T | 67.94 | -0.0272 | 0.0033 | 2.22E-16 |
|  |  | rs645040 | T | G | 53.66 | 0.0293 | 0.004 | 2.39E-13 |
|  |  | rs6831256 | G | A | 54.34 | 0.0258 | 0.0035 | 1.69E-13 |
|  |  | rs6908994 | T | C | 69.69 | 0.0384 | 0.0046 | 1.00E-200 |
|  |  | rs6968554 | G | A | 33.31 | 0.0202 | 0.0035 | 7.86E-09 |
|  |  | rs6995541 | G | A | 51.30 | 0.0265 | 0.0037 | 7.94E-13 |
|  |  | rs7033354 | T | C | 31.23 | -0.019 | 0.0034 | 2.29E-08 |
|  |  | rs719726 | T | C | 32.33 | 0.0199 | 0.0035 | 1.30E-08 |
|  |  | rs731839 | A | G | 38.72 | -0.0224 | 0.0036 | 4.90E-10 |
|  |  | rs749671 | A | G | 38.51 | -0.0211 | 0.0034 | 5.44E-10 |
|  |  | rs77917292 | A | G | 72.03 | 0.1341 | 0.0158 | 1.00E-200 |
|  |  | rs7897379 | C | T | 68.94 | -0.0274 | 0.0033 | 1.00E-200 |
|  |  | rs78980904 | C | T | 118.97 | -0.0589 | 0.0054 | 1.00E-200 |
|  |  | rs8077889 | C | A | 36.00 | 0.0252 | 0.0042 | 1.97E-09 |
|  |  | rs998584 | A | C | 62.71 | 0.0293 | 0.0037 | 2.44E-15 |

**Supplementary Table 3** Genetic instruments screened by GWAS to be associated with cholesterol on vestibular vertigo.

| **Exposure** | **Outcome** | **SNP** | **EA** | **OA** | **F-statistics** | **Beta** | **SE** | **p-value** |
| --- | --- | --- | --- | --- | --- | --- | --- | --- |
| **Cholesterol** | **BPV** | rs10085881 | C | T | 77.71 | 0.00641021 | 0.000727168 | 3.90E-19 |
|  |  | rs10096633 | T | C | 85.27 | -0.0089403 | 0.000968179 | 7.50E-20 |
|  |  | rs1042725 | T | C | 31.85 | 0.00365641 | 0.000647915 | 2.00E-08 |
|  |  | rs10504255 | A | G | 58.88 | -0.0052844 | 0.000688655 | 1.10E-14 |
|  |  | rs11591147 | T | G | 357.46 | -0.0475535 | 0.00251519 | 5.10E-81 |
|  |  | rs11601507 | A | C | 37.88 | 0.00767804 | 0.00124753 | 5.20E-10 |
|  |  | rs11621792 | T | C | 33.11 | 0.00379086 | 0.000658759 | 6.80E-09 |
|  |  | rs1169288 | C | A | 80.62 | 0.00629553 | 0.000701132 | 7.10E-19 |
|  |  | rs117733303 | G | A | 169.49 | 0.0312162 | 0.00239775 | 6.50E-40 |
|  |  | rs11858759 | A | G | 68.81 | 0.00588404 | 0.000709326 | 2.80E-16 |
|  |  | rs12151108 | A | G | 701.91 | -0.0265351 | 0.00100157 | 3.40E-156 |
|  |  | rs1216743 | A | G | 32.45 | 0.00413639 | 0.000726161 | 1.20E-08 |
|  |  | rs12509595 | C | T | 32.54 | 0.0040815 | 0.000715494 | 2.60E-09 |
|  |  | rs1260326 | C | T | 255.93 | -0.0106258 | 0.000664199 | 3.30E-57 |
|  |  | rs12740374 | T | G | 584.02 | -0.0187542 | 0.000776044 | 1.40E-130 |
|  |  | rs12916 | C | T | 274.82 | 0.0109365 | 0.000659712 | 6.70E-62 |
|  |  | rs13173241 | A | G | 32.36 | 0.00453483 | 0.000797233 | 1.00E-08 |
|  |  | rs13379043 | C | T | 36.57 | -0.0044434 | 0.000734733 | 1.50E-09 |
|  |  | rs1367117 | A | G | 483.84 | 0.0151916 | 0.000690641 | 4.20E-108 |
|  |  | rs144311893 | T | C | 259.38 | -0.0369826 | 0.0022963 | 8.40E-59 |
|  |  | rs145208519 | T | C | 30.49 | 0.0110167 | 0.00199512 | 3.60E-08 |
|  |  | rs1584063 | A | G | 44.96 | -0.0043643 | 0.000650867 | 1.30E-11 |
|  |  | rs174536 | C | A | 33.78 | -0.0039827 | 0.000685242 | 1.20E-09 |
|  |  | rs17725246 | C | T | 63.42 | 0.00661106 | 0.000830138 | 1.20E-15 |
|  |  | rs1883025 | T | C | 75.87 | -0.0064241 | 0.000737527 | 8.90E-18 |
|  |  | rs193084249 | G | A | 46.41 | 0.0150976 | 0.00221622 | 1.10E-11 |
|  |  | rs2068888 | A | G | 60.95 | -0.0050761 | 0.000650225 | 3.30E-15 |
|  |  | rs2169387 | G | A | 36.18 | 0.00645434 | 0.00107309 | 1.30E-09 |
|  |  | rs2618567 | T | G | 53.39 | -0.0049718 | 0.000680459 | 1.40E-13 |
|  |  | rs2738447 | C | A | 160.31 | 0.00834437 | 0.000659035 | 1.40E-36 |
|  |  | rs28615248 | C | T | 47.14 | 0.00560562 | 0.000816472 | 2.70E-12 |
|  |  | rs2972147 | C | T | 57.17 | 0.00511702 | 0.000676784 | 3.70E-14 |
|  |  | rs34707604 | C | T | 54.46 | 0.00581081 | 0.000787391 | 1.70E-13 |
|  |  | rs35203651 | C | T | 30.58 | 0.00567145 | 0.00102558 | 2.00E-08 |
|  |  | rs360801 | G | A | 32.47 | -0.0038722 | 0.000679576 | 9.50E-09 |
|  |  | rs3918226 | T | C | 37.52 | 0.00750006 | 0.0012245 | 5.10E-10 |
|  |  | rs4299376 | T | G | 263.76 | -0.0112908 | 0.000695213 | 3.00E-60 |
|  |  | rs456598 | A | G | 53.68 | 0.00687697 | 0.000938596 | 1.10E-13 |
|  |  | rs472495 | T | G | 76.03 | 0.00590634 | 0.000677384 | 1.20E-18 |
|  |  | rs4803748 | T | C | 185.02 | -0.0090924 | 0.000668456 | 2.60E-42 |
|  |  | rs4921914 | T | C | 61.65 | -0.0060841 | 0.000774849 | 3.40E-15 |
|  |  | rs577721086 | C | T | 31.51 | 0.00862948 | 0.00153738 | 2.00E-08 |
|  |  | rs58542926 | T | C | 128.61 | -0.0139615 | 0.00123112 | 4.60E-30 |
|  |  | rs588136 | T | C | 42.59 | -0.0051356 | 0.000786926 | 8.50E-11 |
|  |  | rs6093446 | A | G | 49.72 | 0.00501433 | 0.000711138 | 8.30E-13 |
|  |  | rs622871 | G | A | 60.20 | 0.00551676 | 0.000711024 | 2.20E-15 |
|  |  | rs679582 | A | G | 52.44 | -0.0048367 | 0.000667916 | 6.70E-13 |
|  |  | rs6857 | T | C | 902.88 | 0.0260594 | 0.000867261 | 1.00E-200 |
|  |  | rs687339 | T | C | 37.91 | 0.00472978 | 0.000768228 | 5.90E-10 |
|  |  | rs6905288 | A | G | 65.52 | 0.00529521 | 0.000654198 | 2.20E-16 |
|  |  | rs7140110 | C | T | 37.83 | 0.00434657 | 0.000706659 | 1.30E-09 |
|  |  | rs7214799 | T | C | 46.54 | -0.0044466 | 0.000651803 | 2.10E-12 |
|  |  | rs72698371 | G | A | 36.19 | 0.00724079 | 0.00120364 | 1.00E-09 |
|  |  | rs72805692 | G | A | 36.10 | -0.0061954 | 0.0010311 | 1.50E-09 |
|  |  | rs76895963 | G | T | 42.29 | -0.0165229 | 0.00254087 | 2.10E-11 |
|  |  | rs7746081 | A | G | 58.49 | -0.0053703 | 0.00070221 | 2.20E-14 |
|  |  | rs77542162 | G | A | 109.53 | 0.0232886 | 0.0022252 | 1.70E-26 |
|  |  | rs78058190 | A | G | 42.06 | 0.0109303 | 0.0016854 | 9.80E-11 |
|  |  | rs7903146 | T | C | 58.33 | 0.00543539 | 0.000711672 | 6.80E-15 |
|  |  | rs799157 | C | T | 52.00 | -0.0114878 | 0.00159308 | 2.60E-13 |
|  |  | rs8090363 | G | A | 42.40 | 0.00432555 | 0.00066427 | 8.50E-11 |
|  |  | rs8126001 | T | C | 68.88 | -0.0053851 | 0.000648866 | 1.10E-16 |
|  |  | rs907348 | C | T | 29.98 | -0.0036866 | 0.000673327 | 3.70E-08 |
|  |  | rs9389274 | A | G | 30.25 | 0.00424371 | 0.000771541 | 3.20E-08 |
|  |  | rs9442198 | G | A | 35.55 | -0.0042795 | 0.000717698 | 1.00E-09 |
|  |  | rs9534342 | T | C | 30.57 | -0.0035742 | 0.000646473 | 3.10E-08 |
|  |  | rs972283 | G | A | 34.66 | 0.00382459 | 0.00064968 | 1.20E-08 |
| **Cholesterol** | **MD** |  |  |  |  |  |  |  |
|  |  | rs10085881 | T | C | 77.71 | -0.0064102 | 0.000727168 | 3.90E-19 |
|  |  | rs10096633 | C | T | 85.27 | 0.00894032 | 0.000968179 | 7.50E-20 |
|  |  | rs1042725 | C | T | 31.85 | -0.0036564 | 0.000647915 | 2.00E-08 |
|  |  | rs10504255 | G | A | 58.88 | 0.00528436 | 0.000688655 | 1.10E-14 |
|  |  | rs11591147 | G | T | 357.46 | 0.0475535 | 0.00251519 | 5.10E-81 |
|  |  | rs11601507 | C | A | 37.88 | -0.007678 | 0.00124753 | 5.20E-10 |
|  |  | rs11621792 | C | T | 33.11 | -0.0037909 | 0.000658759 | 6.80E-09 |
|  |  | rs1169288 | A | C | 80.62 | -0.0062955 | 0.000701132 | 7.10E-19 |
|  |  | rs117733303 | A | G | 169.49 | -0.0312162 | 0.00239775 | 6.50E-40 |
|  |  | rs11858759 | G | A | 68.81 | -0.005884 | 0.000709326 | 2.80E-16 |
|  |  | rs12151108 | G | A | 701.91 | 0.0265351 | 0.00100157 | 3.40E-156 |
|  |  | rs1216743 | G | A | 32.45 | -0.0041364 | 0.000726161 | 1.20E-08 |
|  |  | rs1260326 | T | C | 255.93 | 0.0106258 | 0.000664199 | 3.30E-57 |
|  |  | rs12740374 | G | T | 584.02 | 0.0187542 | 0.000776044 | 1.40E-130 |
|  |  | rs12916 | T | C | 274.82 | -0.0109365 | 0.000659712 | 6.70E-62 |
|  |  | rs13173241 | G | A | 32.36 | -0.0045348 | 0.000797233 | 1.00E-08 |
|  |  | rs13379043 | T | C | 36.57 | 0.00444335 | 0.000734733 | 1.50E-09 |
|  |  | rs1367117 | G | A | 483.84 | -0.0151916 | 0.000690641 | 4.20E-108 |
|  |  | rs144311893 | C | T | 259.38 | 0.0369826 | 0.0022963 | 8.40E-59 |
|  |  | rs145208519 | C | T | 30.49 | -0.0110167 | 0.00199512 | 3.60E-08 |
|  |  | rs147233090 | C | T | 34.07 | -0.0124289 | 0.00212946 | 8.00E-09 |
|  |  | rs1584063 | G | A | 44.96 | 0.0043643 | 0.000650867 | 1.30E-11 |
|  |  | rs17725246 | T | C | 63.42 | -0.0066111 | 0.000830138 | 1.20E-15 |
|  |  | rs1883025 | C | T | 75.87 | 0.0064241 | 0.000737527 | 8.90E-18 |
|  |  | rs193084249 | A | G | 46.41 | -0.0150976 | 0.00221622 | 1.10E-11 |
|  |  | rs2068888 | G | A | 60.95 | 0.00507614 | 0.000650225 | 3.30E-15 |
|  |  | rs2169387 | A | G | 36.18 | -0.0064543 | 0.00107309 | 1.30E-09 |
|  |  | rs2618567 | G | T | 53.39 | 0.00497182 | 0.000680459 | 1.40E-13 |
|  |  | rs2738447 | A | C | 160.31 | -0.0083444 | 0.000659035 | 1.40E-36 |
|  |  | rs28615248 | T | C | 47.14 | -0.0056056 | 0.000816472 | 2.70E-12 |
|  |  | rs2972147 | T | C | 57.17 | -0.005117 | 0.000676784 | 3.70E-14 |
|  |  | rs34707604 | T | C | 54.46 | -0.0058108 | 0.000787391 | 1.70E-13 |
|  |  | rs35203651 | T | C | 30.58 | -0.0056715 | 0.00102558 | 2.00E-08 |
|  |  | rs360801 | A | G | 32.47 | 0.00387215 | 0.000679576 | 9.50E-09 |
|  |  | rs3918226 | C | T | 37.52 | -0.0075001 | 0.0012245 | 5.10E-10 |
|  |  | rs4299376 | G | T | 263.76 | 0.0112908 | 0.000695213 | 3.00E-60 |
|  |  | rs456598 | G | A | 53.68 | -0.006877 | 0.000938596 | 1.10E-13 |
|  |  | rs472495 | G | T | 76.03 | -0.0059063 | 0.000677384 | 1.20E-18 |
|  |  | rs4803748 | C | T | 185.02 | 0.00909238 | 0.000668456 | 2.60E-42 |
|  |  | rs4921914 | C | T | 61.65 | 0.00608414 | 0.000774849 | 3.40E-15 |
|  |  | rs556107 | C | T | 60.37 | -0.0050676 | 0.000652202 | 1.10E-14 |
|  |  | rs577721086 | T | C | 31.51 | -0.0086295 | 0.00153738 | 2.00E-08 |
|  |  | rs58542926 | C | T | 128.61 | 0.0139615 | 0.00123112 | 4.60E-30 |
|  |  | rs588136 | C | T | 42.59 | 0.00513555 | 0.000786926 | 8.50E-11 |
|  |  | rs6093446 | G | A | 49.72 | -0.0050143 | 0.000711138 | 8.30E-13 |
|  |  | rs622871 | A | G | 60.20 | -0.0055168 | 0.000711024 | 2.20E-15 |
|  |  | rs679582 | G | A | 52.44 | 0.00483667 | 0.000667916 | 6.70E-13 |
|  |  | rs6857 | C | T | 902.88 | -0.0260594 | 0.000867261 | 1.00E-200 |
|  |  | rs687339 | C | T | 37.91 | -0.0047298 | 0.000768228 | 5.90E-10 |
|  |  | rs6905288 | G | A | 65.52 | -0.0052952 | 0.000654198 | 2.20E-16 |
|  |  | rs7140110 | T | C | 37.83 | -0.0043466 | 0.000706659 | 1.30E-09 |
|  |  | rs7214799 | C | T | 46.54 | 0.00444658 | 0.000651803 | 2.10E-12 |
|  |  | rs72698371 | A | G | 36.19 | -0.0072408 | 0.00120364 | 1.00E-09 |
|  |  | rs72805692 | A | G | 36.10 | 0.00619544 | 0.0010311 | 1.50E-09 |
|  |  | rs72837687 | G | A | 35.59 | 0.00487029 | 0.000816337 | 2.10E-09 |
|  |  | rs7581601 | A | C | 31.15 | 0.00394211 | 0.000706276 | 2.70E-08 |
|  |  | rs76895963 | T | G | 42.29 | 0.0165229 | 0.00254087 | 2.10E-11 |
|  |  | rs7746081 | G | A | 58.49 | 0.00537028 | 0.00070221 | 2.20E-14 |
|  |  | rs77542162 | A | G | 109.53 | -0.0232886 | 0.0022252 | 1.70E-26 |
|  |  | rs78058190 | G | A | 42.06 | -0.0109303 | 0.0016854 | 9.80E-11 |
|  |  | rs7903146 | C | T | 58.33 | -0.0054354 | 0.000711672 | 6.80E-15 |
|  |  | rs799157 | T | C | 52.00 | 0.0114878 | 0.00159308 | 2.60E-13 |
|  |  | rs8090363 | A | G | 42.40 | -0.0043256 | 0.00066427 | 8.50E-11 |
|  |  | rs8126001 | C | T | 68.88 | 0.00538509 | 0.000648866 | 1.10E-16 |
|  |  | rs907348 | T | C | 29.98 | 0.00368655 | 0.000673327 | 3.70E-08 |
|  |  | rs9389274 | G | A | 30.25 | -0.0042437 | 0.000771541 | 3.20E-08 |
|  |  | rs9442198 | A | G | 35.55 | 0.00427947 | 0.000717698 | 1.00E-09 |
|  |  | rs9534342 | C | T | 30.57 | 0.00357418 | 0.000646473 | 3.10E-08 |
|  |  | rs972283 | A | G | 34.66 | -0.0038246 | 0.00064968 | 1.20E-08 |
| **Cholesterol** | **VN** |  |  |  |  |  |  |  |
|  |  | rs10085881 | T | C | 77.71 | -0.0064102 | 0.000727168 | 3.90E-19 |
|  |  | rs10096633 | C | T | 85.27 | 0.00894032 | 0.000968179 | 7.50E-20 |
|  |  | rs1042725 | C | T | 31.85 | -0.0036564 | 0.000647915 | 2.00E-08 |
|  |  | rs10504255 | G | A | 58.88 | 0.00528436 | 0.000688655 | 1.10E-14 |
|  |  | rs11601507 | C | A | 37.88 | -0.007678 | 0.00124753 | 5.20E-10 |
|  |  | rs11621792 | C | T | 33.11 | -0.0037909 | 0.000658759 | 6.80E-09 |
|  |  | rs1169288 | A | C | 80.62 | -0.0062955 | 0.000701132 | 7.10E-19 |
|  |  | rs117733303 | A | G | 169.49 | -0.0312162 | 0.00239775 | 6.50E-40 |
|  |  | rs11858759 | G | A | 68.81 | -0.005884 | 0.000709326 | 2.80E-16 |
|  |  | rs12151108 | G | A | 701.91 | 0.0265351 | 0.00100157 | 3.40E-156 |
|  |  | rs1216743 | G | A | 32.45 | -0.0041364 | 0.000726161 | 1.20E-08 |
|  |  | rs12509595 | T | C | 32.54 | -0.0040815 | 0.000715494 | 2.60E-09 |
|  |  | rs1260326 | T | C | 255.93 | 0.0106258 | 0.000664199 | 3.30E-57 |
|  |  | rs12740374 | G | T | 584.02 | 0.0187542 | 0.000776044 | 1.40E-130 |
|  |  | rs12916 | T | C | 274.82 | -0.0109365 | 0.000659712 | 6.70E-62 |
|  |  | rs13173241 | G | A | 32.36 | -0.0045348 | 0.000797233 | 1.00E-08 |
|  |  | rs13379043 | T | C | 36.57 | 0.00444335 | 0.000734733 | 1.50E-09 |
|  |  | rs1367117 | G | A | 483.84 | -0.0151916 | 0.000690641 | 4.20E-108 |
|  |  | rs144311893 | C | T | 259.38 | 0.0369826 | 0.0022963 | 8.40E-59 |
|  |  | rs145208519 | C | T | 30.49 | -0.0110167 | 0.00199512 | 3.60E-08 |
|  |  | rs1584063 | G | A | 44.96 | 0.0043643 | 0.000650867 | 1.30E-11 |
|  |  | rs17725246 | T | C | 63.42 | -0.0066111 | 0.000830138 | 1.20E-15 |
|  |  | rs1883025 | C | T | 75.87 | 0.0064241 | 0.000737527 | 8.90E-18 |
|  |  | rs193084249 | A | G | 46.41 | -0.0150976 | 0.00221622 | 1.10E-11 |
|  |  | rs2169387 | A | G | 36.18 | -0.0064543 | 0.00107309 | 1.30E-09 |
|  |  | rs2618567 | G | T | 53.39 | 0.00497182 | 0.000680459 | 1.40E-13 |
|  |  | rs2738447 | A | C | 160.31 | -0.0083444 | 0.000659035 | 1.40E-36 |
|  |  | rs28615248 | T | C | 47.14 | -0.0056056 | 0.000816472 | 2.70E-12 |
|  |  | rs2972147 | T | C | 57.17 | -0.005117 | 0.000676784 | 3.70E-14 |
|  |  | rs34707604 | T | C | 54.46 | -0.0058108 | 0.000787391 | 1.70E-13 |
|  |  | rs35203651 | T | C | 30.58 | -0.0056715 | 0.00102558 | 2.00E-08 |
|  |  | rs360801 | A | G | 32.47 | 0.00387215 | 0.000679576 | 9.50E-09 |
|  |  | rs3918226 | C | T | 37.52 | -0.0075001 | 0.0012245 | 5.10E-10 |
|  |  | rs4299376 | G | T | 263.76 | 0.0112908 | 0.000695213 | 3.00E-60 |
|  |  | rs456598 | G | A | 53.68 | -0.006877 | 0.000938596 | 1.10E-13 |
|  |  | rs472495 | G | T | 76.03 | -0.0059063 | 0.000677384 | 1.20E-18 |
|  |  | rs4803748 | C | T | 185.02 | 0.00909238 | 0.000668456 | 2.60E-42 |
|  |  | rs4921914 | C | T | 61.65 | 0.00608414 | 0.000774849 | 3.40E-15 |
|  |  | rs556107 | C | T | 60.37 | -0.0050676 | 0.000652202 | 1.10E-14 |
|  |  | rs577721086 | T | C | 31.51 | -0.0086295 | 0.00153738 | 2.00E-08 |
|  |  | rs58542926 | C | T | 128.61 | 0.0139615 | 0.00123112 | 4.60E-30 |
|  |  | rs6093446 | G | A | 49.72 | -0.0050143 | 0.000711138 | 8.30E-13 |
|  |  | rs622871 | A | G | 60.20 | -0.0055168 | 0.000711024 | 2.20E-15 |
|  |  | rs679582 | G | A | 52.44 | 0.00483667 | 0.000667916 | 6.70E-13 |
|  |  | rs687339 | C | T | 37.91 | -0.0047298 | 0.000768228 | 5.90E-10 |
|  |  | rs6905288 | G | A | 65.52 | -0.0052952 | 0.000654198 | 2.20E-16 |
|  |  | rs7140110 | T | C | 37.83 | -0.0043466 | 0.000706659 | 1.30E-09 |
|  |  | rs7214799 | C | T | 46.54 | 0.00444658 | 0.000651803 | 2.10E-12 |
|  |  | rs72805692 | A | G | 36.10 | 0.00619544 | 0.0010311 | 1.50E-09 |
|  |  | rs72837687 | G | A | 35.59 | 0.00487029 | 0.000816337 | 2.10E-09 |
|  |  | rs7581601 | A | C | 31.15 | 0.00394211 | 0.000706276 | 2.70E-08 |
|  |  | rs76895963 | T | G | 42.29 | 0.0165229 | 0.00254087 | 2.10E-11 |
|  |  | rs7746081 | G | A | 58.49 | 0.00537028 | 0.00070221 | 2.20E-14 |
|  |  | rs78058190 | G | A | 42.06 | -0.0109303 | 0.0016854 | 9.80E-11 |
|  |  | rs7903146 | C | T | 58.33 | -0.0054354 | 0.000711672 | 6.80E-15 |
|  |  | rs799157 | T | C | 52.00 | 0.0114878 | 0.00159308 | 2.60E-13 |
|  |  | rs8090363 | A | G | 42.40 | -0.0043256 | 0.00066427 | 8.50E-11 |
|  |  | rs8126001 | C | T | 68.88 | 0.00538509 | 0.000648866 | 1.10E-16 |
|  |  | rs907348 | T | C | 29.98 | 0.00368655 | 0.000673327 | 3.70E-08 |
|  |  | rs9389274 | G | A | 30.25 | -0.0042437 | 0.000771541 | 3.20E-08 |
|  |  | rs972283 | A | G | 34.66 | -0.0038246 | 0.00064968 | 1.20E-08 |
| **Cholesterol** | **VD** |  |  |  |  |  |  |  |
|  |  | rs10085881 | T | C | 77.71 | -0.0064102 | 0.000727168 | 3.90E-19 |
|  |  | rs10096633 | C | T | 85.27 | 0.00894032 | 0.000968179 | 7.50E-20 |
|  |  | rs1042725 | C | T | 31.85 | -0.0036564 | 0.000647915 | 2.00E-08 |
|  |  | rs10504255 | G | A | 58.88 | 0.00528436 | 0.000688655 | 1.10E-14 |
|  |  | rs11591147 | G | T | 357.46 | 0.0475535 | 0.00251519 | 5.10E-81 |
|  |  | rs11601507 | C | A | 37.88 | -0.007678 | 0.00124753 | 5.20E-10 |
|  |  | rs11621792 | C | T | 33.11 | -0.0037909 | 0.000658759 | 6.80E-09 |
|  |  | rs1169288 | A | C | 80.62 | -0.0062955 | 0.000701132 | 7.10E-19 |
|  |  | rs117733303 | A | G | 169.49 | -0.0312162 | 0.00239775 | 6.50E-40 |
|  |  | rs11858759 | G | A | 68.81 | -0.005884 | 0.000709326 | 2.80E-16 |
|  |  | rs12151108 | G | A | 701.91 | 0.0265351 | 0.00100157 | 3.40E-156 |
|  |  | rs1216743 | G | A | 32.45 | -0.0041364 | 0.000726161 | 1.20E-08 |
|  |  | rs12509595 | T | C | 32.54 | -0.0040815 | 0.000715494 | 2.60E-09 |
|  |  | rs1260326 | T | C | 255.93 | 0.0106258 | 0.000664199 | 3.30E-57 |
|  |  | rs12916 | T | C | 274.82 | -0.0109365 | 0.000659712 | 6.70E-62 |
|  |  | rs13173241 | G | A | 32.36 | -0.0045348 | 0.000797233 | 1.00E-08 |
|  |  | rs13379043 | T | C | 36.57 | 0.00444335 | 0.000734733 | 1.50E-09 |
|  |  | rs1367117 | G | A | 483.84 | -0.0151916 | 0.000690641 | 4.20E-108 |
|  |  | rs144311893 | C | T | 259.38 | 0.0369826 | 0.0022963 | 8.40E-59 |
|  |  | rs145208519 | C | T | 30.49 | -0.0110167 | 0.00199512 | 3.60E-08 |
|  |  | rs147233090 | C | T | 34.07 | -0.0124289 | 0.00212946 | 8.00E-09 |
|  |  | rs1584063 | G | A | 44.96 | 0.0043643 | 0.000650867 | 1.30E-11 |
|  |  | rs17725246 | T | C | 63.42 | -0.0066111 | 0.000830138 | 1.20E-15 |
|  |  | rs1883025 | C | T | 75.87 | 0.0064241 | 0.000737527 | 8.90E-18 |
|  |  | rs193084249 | A | G | 46.41 | -0.0150976 | 0.00221622 | 1.10E-11 |
|  |  | rs2068888 | G | A | 60.95 | 0.00507614 | 0.000650225 | 3.30E-15 |
|  |  | rs2169387 | A | G | 36.18 | -0.0064543 | 0.00107309 | 1.30E-09 |
|  |  | rs2618567 | G | T | 53.39 | 0.00497182 | 0.000680459 | 1.40E-13 |
|  |  | rs2738447 | A | C | 160.31 | -0.0083444 | 0.000659035 | 1.40E-36 |
|  |  | rs28615248 | T | C | 47.14 | -0.0056056 | 0.000816472 | 2.70E-12 |
|  |  | rs2972147 | T | C | 57.17 | -0.005117 | 0.000676784 | 3.70E-14 |
|  |  | rs34707604 | T | C | 54.46 | -0.0058108 | 0.000787391 | 1.70E-13 |
|  |  | rs35203651 | T | C | 30.58 | -0.0056715 | 0.00102558 | 2.00E-08 |
|  |  | rs360801 | A | G | 32.47 | 0.00387215 | 0.000679576 | 9.50E-09 |
|  |  | rs3918226 | C | T | 37.52 | -0.0075001 | 0.0012245 | 5.10E-10 |
|  |  | rs4299376 | G | T | 263.76 | 0.0112908 | 0.000695213 | 3.00E-60 |
|  |  | rs456598 | G | A | 53.68 | -0.006877 | 0.000938596 | 1.10E-13 |
|  |  | rs472495 | G | T | 76.03 | -0.0059063 | 0.000677384 | 1.20E-18 |
|  |  | rs4803748 | C | T | 185.02 | 0.00909238 | 0.000668456 | 2.60E-42 |
|  |  | rs4921914 | C | T | 61.65 | 0.00608414 | 0.000774849 | 3.40E-15 |
|  |  | rs556107 | C | T | 60.37 | -0.0050676 | 0.000652202 | 1.10E-14 |
|  |  | rs577721086 | T | C | 31.51 | -0.0086295 | 0.00153738 | 2.00E-08 |
|  |  | rs58542926 | C | T | 128.61 | 0.0139615 | 0.00123112 | 4.60E-30 |
|  |  | rs588136 | C | T | 42.59 | 0.00513555 | 0.000786926 | 8.50E-11 |
|  |  | rs6093446 | G | A | 49.72 | -0.0050143 | 0.000711138 | 8.30E-13 |
|  |  | rs622871 | A | G | 60.20 | -0.0055168 | 0.000711024 | 2.20E-15 |
|  |  | rs6857 | C | T | 902.88 | -0.0260594 | 0.000867261 | 1.00E-200 |
|  |  | rs687339 | C | T | 37.91 | -0.0047298 | 0.000768228 | 5.90E-10 |
|  |  | rs6905288 | G | A | 65.52 | -0.0052952 | 0.000654198 | 2.20E-16 |
|  |  | rs7140110 | T | C | 37.83 | -0.0043466 | 0.000706659 | 1.30E-09 |
|  |  | rs7214799 | C | T | 46.54 | 0.00444658 | 0.000651803 | 2.10E-12 |
|  |  | rs72698371 | A | G | 36.19 | -0.0072408 | 0.00120364 | 1.00E-09 |
|  |  | rs72805692 | A | G | 36.10 | 0.00619544 | 0.0010311 | 1.50E-09 |
|  |  | rs72837687 | G | A | 35.59 | 0.00487029 | 0.000816337 | 2.10E-09 |
|  |  | rs76895963 | T | G | 42.29 | 0.0165229 | 0.00254087 | 2.10E-11 |
|  |  | rs7746081 | G | A | 58.49 | 0.00537028 | 0.00070221 | 2.20E-14 |
|  |  | rs77542162 | A | G | 109.53 | -0.0232886 | 0.0022252 | 1.70E-26 |
|  |  | rs78058190 | G | A | 42.06 | -0.0109303 | 0.0016854 | 9.80E-11 |
|  |  | rs7903146 | C | T | 58.33 | -0.0054354 | 0.000711672 | 6.80E-15 |
|  |  | rs799157 | T | C | 52.00 | 0.0114878 | 0.00159308 | 2.60E-13 |
|  |  | rs8090363 | A | G | 42.40 | -0.0043256 | 0.00066427 | 8.50E-11 |
|  |  | rs8126001 | C | T | 68.88 | 0.00538509 | 0.000648866 | 1.10E-16 |
|  |  | rs907348 | T | C | 29.98 | 0.00368655 | 0.000673327 | 3.70E-08 |
|  |  | rs9389274 | G | A | 30.25 | -0.0042437 | 0.000771541 | 3.20E-08 |
|  |  | rs9442198 | A | G | 35.55 | 0.00427947 | 0.000717698 | 1.00E-09 |
|  |  | rs9534342 | C | T | 30.57 | 0.00357418 | 0.000646473 | 3.10E-08 |
|  |  | rs972283 | A | G | 34.66 | -0.0038246 | 0.00064968 | 1.20E-08 |

**Supplementary Table 4** The results of heterogeneity test.

| **Exposure** | **Outcome** | **Method** | **Cochrane’s Q** | **Cochrane’s *Q*_pval** |
| --- | --- | --- | --- | --- |
| Triglyceride | BPV | MR Egger | 43.37632 | 0.8910551 |
|  |  | Inverse variance weighted | 44.38611 | 0.8884174 |
| Triglyceride | MD | MR Egger | 51.74519 | 0.7055804 |
|  |  | Inverse variance weighted | 54.6141 | 0.637734 |
| Triglyceride | VN | MR Egger | 78.61596 | 0.05376908 |
|  |  | Inverse variance weighted | 78.62372 | 0.0639281 |
| Triglyceride | VD | MR Egger | 73.25975 | 0.07223434 |
|  |  | Inverse variance weighted | 75.60862 | 0.06006225 |
| Cholesterol | BPV | MR Egger | 41.49694 | 0.9898297 |
|  |  | Inverse variance weighted | 42.28694 | 0.9898525 |
| Cholesterol | MD | MR Egger | 75.66806 | 0.218937 |
|  |  | Inverse variance weighted | 75.82268 | 0.2409205 |
| Cholesterol | VN | MR Egger | 55.19083 | 0.6166713 |
|  |  | Inverse variance weighted | 58.15233 | 0.5435448 |
| Cholesterol | VD | MR Egger | 54.20943 | 0.8277073 |
|  |  | Inverse variance weighted | 54.59278 | 0.840785 |

**Supplementary Table 5** The results of pleiotropy test.

| **Exposure** | **Outcome** | **egger_intercept** | **SE** | **Pleiotropy pval** |
| --- | --- | --- | --- | --- |
| Triglyceride | BPV | 0.005508087 | 0.005481307 | 0.3192744 |
| Triglyceride | MD | 0.01576866 | 0.009309707 | 0.09567022 |
| Triglyceride | VN | 0.000865795 | 0.01124609 | 0.9388906 |
| Triglyceride | VD | 0.006351491 | 0.004698301 | 0.1817573 |
| Cholesterol | BPV | 0.003468509 | 0.003902384 | 0.3773784 |
| Cholesterol | MD | -0.002652822 | 0.007169528 | 0.7125396 |
| Cholesterol | VN | -0.01497152 | 0.008699814 | 0.09050955 |
| Cholesterol | VD | 0.001866236 | 0.003014162 | 0.5379778 |

**Supplementary Table 6** MVMR estimates for the causal associations of triglyceride with the risk of BPV, VN, and VD.

| **Exposure** | **Outcome** | **Adjusted for** | **Beta** | **95% CI** | **SE** | **p-value** |
| --- | --- | --- | --- | --- | --- | --- |
| Triglyceride | BPV | BMI | 0.136 | 0.021, 0.251 | 0.059 | 0.021 |
|  |  | hypertension | 0.097 | 0.006, 0.188 | 0.046 | 0.037 |
|  |  | Type 2 diabetes | 0.110 | 0.007, 0.213 | 0.053 | 0.035 |
|  |  | Vitamin D | 0.083 | -0.024, 0.189 | 0.055 | 0.129 |
| Triglyceride | VN | BMI | 0.249 | 0.051, 0.446 | 0.101 | 0.013 |
|  |  | Hypertension | 0.262 | 0.057, 0.467 | 0.105 | 0.012 |
|  |  | Type 2 diabetes | 0.256 | 0.041, 0.471 | 0.110 | 0.019 |
|  |  | Vitamin D | 0.275 | 0.043, 0.505 | 0.118 | 0.019 |
| Triglyceride | VD | BMI | 0.097 | 0.006, 0.186 | 0.046 | 0.036 |
|  |  | hypertension | 0.089 | 0.001, 0.175 | 0.045 | 0.047 |
|  |  | Type 2 diabetes | 0.103 | 0.014, 0.191 | 0.045 | 0.023 |
|  |  | Vitamin D | 0.072 | -0.025, 0.168 | 0.049 | 0.146 |

**Supplementary Table 7** MVMR estimates for the causal associations of cholesterol with the risk of BPV, VN, and VD.

| **Exposure** | **Outcome** | **Adjusted for** | **Beta** | **95% CI** | **SE** | **p-value** |
| --- | --- | --- | --- | --- | --- | --- |
| Cholesterol | BPV | BMI | 0.630 | 0.012, 1.247 | 0.315 | 0.045 |
|  |  | Hypertension | 0.618 | 0.057, 1.178 | 0.286 | 0.031 |
|  |  | Type 2 diabetes | 0.569 | 0.031, 1.106 | 0.274 | 0.037 |
|  |  | Vitamin D | 0.431 | -0.148, 1.010 | 0.296 | 0.145 |
| Cholesterol | VN | BMI | 1.181 | 0.130, 2.231 | 0.536 | 0.028 |
|  |  | hypertension | 1.551 | 0.403, 2.699 | 0.586 | 0.008 |
|  |  | Type 2 diabetes | 1.671 | 0.551, 2.791 | 0.572 | 0.003 |
|  |  | Vitamin D | 0.907 | -0.339, 2.153 | 0.636 | 0.154 |
| Cholesterol | VD | BMI | 0.545 | 0.080, 1.009 | 0.237 | 0.022 |
|  |  | hypertension | 0.564 | 0.098, 1.029 | 0.238 | 0.018 |
|  |  | Type 2 diabetes | 0.578 | 0.139, 1.016 | 0.224 | 0.009 |
|  |  | Vitamin D | 0.414 | -0.038, 0.866 | 0.231 | 0.073 |

**Supplementary Table 8** The causal effect of triglyceride on BMI, hypertension, type 2 diabetes, and vitamin D.

| **Exposure** | **Outcome** | **Beta** | **95% CI** | **SE** | **p-value** |
| --- | --- | --- | --- | --- | --- |
| Triglyceride | BMI | 0.039 | 0.001, 0.078 | 0.020 | 0.042 |
| BMI | Triglyceride | -0.193 | -0.238, -0.148 | 0.023 | 3.04E-17 |
| Triglyceride | hypertension | 0.000 | -0.0004, 0.0012 | 0.000 | 0.321 |
| Triglyceride | Type 2 diabetes | 0.010 | 0.002, 0.017 | 0.010 | 0.009 |
| Type 2 diabetes | Triglyceride | 0.476 | 0.029, 0.923 | 0.228 | 0.037 |
| Triglyceride | Vitamin D | -0.116 | -0.149, -0.083 | 0.016 | 4.13E-12 |
| Vitamin D | Triglyceride | -0.414 | -0.659, -0.169 | 0.124 | 9.17E-04 |

**Supplementary Table 9** The causal effect of cholesterol on BMI, hypertension, type 2 diabetes, vitamin D.

| **Exposure** | **Outcome** | **Beta** | **95% CI** | **SE** | **p-value** |
| --- | --- | --- | --- | --- | --- |
| Cholesterol | BMI | 0.273 | 0.147, 0.399 | 0.064 | 0.0000195 |
| BMI | Cholesterol | -0.012 | -0.238, -0.148 | 0.002 | 0.0000165 |
| Cholesterol | hypertension | 0.0000455 | -0.002, 0.002 | 0.001 | 0.9758227 |
| Cholesterol | Type 2 diabetes | 0.074 | 0.042, 0.105 | 0.016 | 0.00000368 |
| Type 2 diabetes | Cholesterol | 0.358 | 0.294, 0.421 | 0.032 | 2.65E-28 |
| Cholesterol | Vitamin D | -0.801 | -0.941, -0.659 | 0.071 | 7.24E-29 |
| Vitamin D | Cholesterol | -0.011 | -0.025, 0.003 | 0.007 | 0.137 |

**Supplementary Table 10** The causal effect of vitamin D on BPV, VN, and VD.

| **Exposure** | **Outcome** | **Beta** | **95% CI** | **SE** | **p-value** |
| --- | --- | --- | --- | --- | --- |
| Vitamin D | BPV | -0.216 | -0.409, -0.024 | 0.098 | 0.027 |
| Vitamin D | VN | -0.368 | -0.676, -0.059 | 0.157 | 0.019 |
| Vitamin D | VD | -0.177 | -0.304, -0.051 | 0.064 | 0.006 |

**Supplementary Figure 1** Leave-one-out sensitivity analysis of causal associations between triglyceride and vestibular vertigo. (A) Benign paroxysmal vertigo, (B) Meniere's disease, (C) Vestibular neuronitis, (D) Vestibular dysfunction.


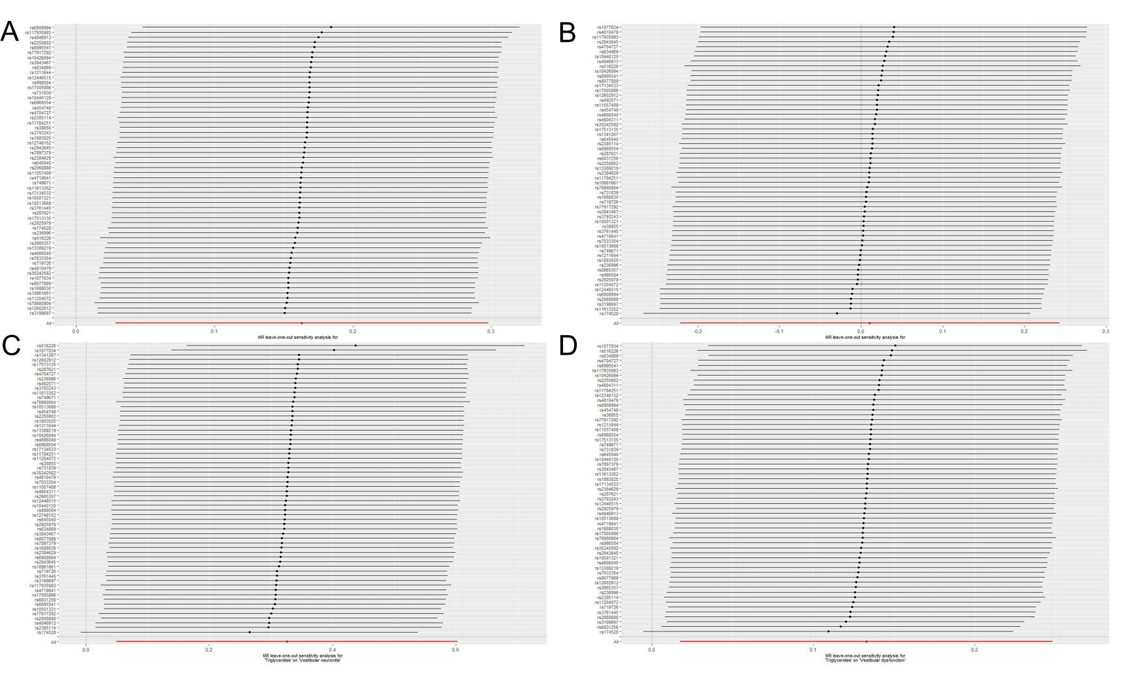


**Supplementary Figure 2** Leave-one-out sensitivity analysis of causal associations between cholesterol and vestibular vertigo. (A) Benign paroxysmal vertigo, (B) Meniere's disease, (C) Vestibular neuronitis, (D) Vestibular dysfunction.


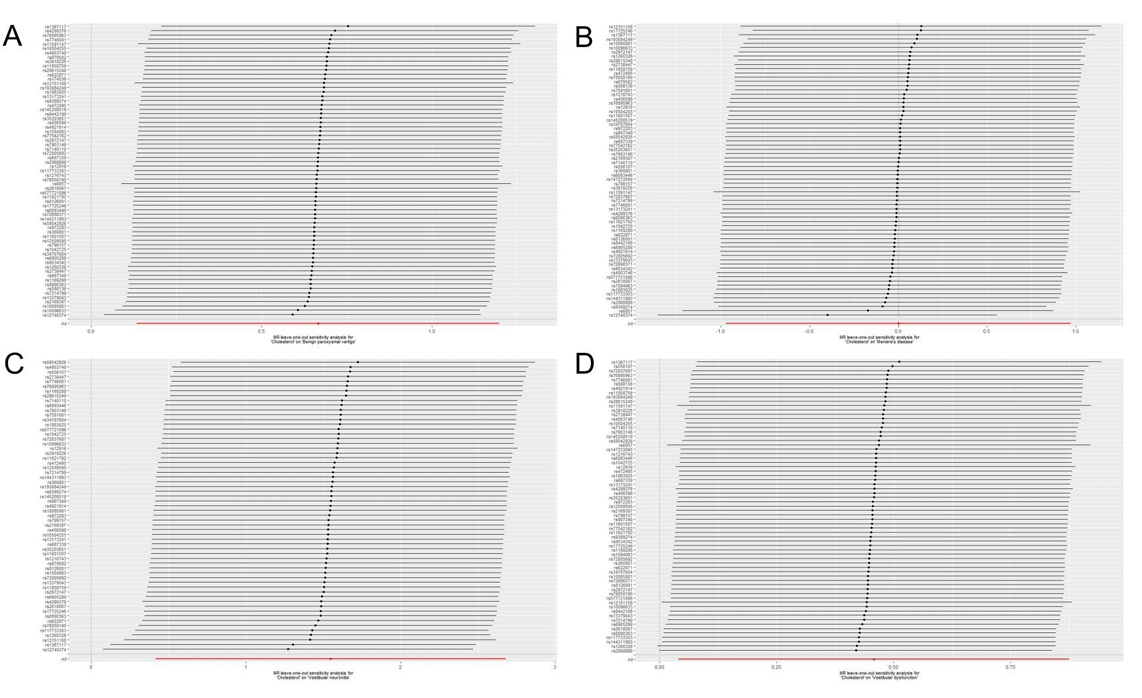

Supplement: Supplementary file 1 [file mmc1.docx]
